# The Glomerular Endothelium Restricts Albumin Filtration

**DOI:** 10.3389/fmed.2021.766689

**Published:** 2021-11-29

**Authors:** Barbara J. Ballermann, Jenny Nyström, Börje Haraldsson

**Affiliations:** ^1^Department of Medicine, University of Alberta, Edmonton, AB, Canada; ^2^Institute of Neuroscience and Physiology, University of Gothenburg, Gothenburg, Sweden

**Keywords:** endothelial surface layer, endothelial dysfunction, fenestrae, glycocalyx, hyaluronan, permselectivity, proteoglycans, thrombotic microangiopathy

## Abstract

Inflammatory activation and/or dysfunction of the glomerular endothelium triggers proteinuria in many systemic and localized vascular disorders. Among them are the thrombotic microangiopathies, many forms of glomerulonephritis, and acute inflammatory episodes like sepsis and COVID-19 illness. Another example is the chronic endothelial dysfunction that develops in cardiovascular disease and in metabolic disorders like diabetes. While the glomerular endothelium is a porous sieve that filters prodigious amounts of water and small solutes, it also bars the bulk of albumin and large plasma proteins from passing into the glomerular filtrate. This endothelial barrier function is ascribed predominantly to the endothelial glycocalyx with its endothelial surface layer, that together form a relatively thick, mucinous coat composed of glycosaminoglycans, proteoglycans, glycolipids, sialomucins and other glycoproteins, as well as secreted and circulating proteins. The glycocalyx/endothelial surface layer not only covers the glomerular endothelium; it extends into the endothelial fenestrae. Some glycocalyx components span or are attached to the apical endothelial cell plasma membrane and form the formal glycocalyx. Other components, including small proteoglycans and circulating proteins like albumin and orosomucoid, form the endothelial surface layer and are bound to the glycocalyx due to weak intermolecular interactions. Indeed, bound plasma albumin is a major constituent of the endothelial surface layer and contributes to its barrier function. A role for glomerular endothelial cells in the barrier of the glomerular capillary wall to protein filtration has been demonstrated by many elegant studies. However, it can only be fully understood in the context of other components, including the glomerular basement membrane, the podocytes and reabsorption of proteins by tubule epithelial cells. Discovery of the precise mechanisms that lead to glycocalyx/endothelial surface layer disruption within glomerular capillaries will hopefully lead to pharmacological interventions that specifically target this important structure.

## Introduction

Albuminuria is the hallmark of essentially all disorders affecting renal glomeruli. In some cases, endothelial cell (EC) injury predominates, for instance the hemolytic uremic syndrome (HUS) (1), thrombotic thrombocytopenic purpura (TTP) (2), pre-eclampsia (3, 4), and nephrotoxicity due to VEGF inhibitors (5). Transient proteinuria also accompanies generalized EC activation, in the presence of sepsis (6) or viral infections (7, 8). Microalbuminuria is a feature of widespread EC dysfunction in diabetes (9, 10) and in cardiovascular disease, where it is a predictor of cardiovascular risk (11–19). EC activation and injury also contributes to proteinuria in many glomerulonephritides and vasculitides affecting glomeruli. This review will tackle the question to what extent the glomerular endothelium contributes to the glomerular capillary wall (GCW) barrier preventing filtration of albumin and other circulating macromolecules. It will review the molecular components of this part of the glomerular capillary barrier (GCB) followed by an exploration of some human diseases in which glomerular EC injury or dysfunction leads to proteinuria. Due to space limitations components of the endothelial glycocalyx that govern complement activation, coagulation and inflammatory cell adhesion and transmigration will not be reviewed in detail.

It is useful to recall that without a glomerular barrier to macromolecule filtration, the potential filtered load of albumin would be 3–5 g/min (~4–7 kg/24 h) in human adults, assuming a plasma albumin concentration of 40 g/L and a glomerular filtration rate (GFR) in the range of 75–125 ml/min. Given the upper limit for urinary albumin excretion of 30 mg/24 h in normal adults, it follows that <0.001% (~ 1/100,000) of the potential filtered load of albumin is excreted in the urine. Indeed, even with severe nephrotic syndrome, urinary albumin loss usually represents <1% of the potential filtered load. Thus, extraordinarily effective mechanisms prevent urinary loss of albumin and other circulating macromolecules.

## The Glomerular Capillary Wall Barrier

### The Glomerular Sieving Coefficient (θ) for Albumin (_g_θ_Alb_) and Other Macromolecules

The GCW sieving coefficient, _g_θ, is defined as the ratio of the Bowman's space to plasma concentration for any given molecule. Since the GCW prevents macromolecule filtration despite its large hydraulic conductivity (water permeability), _g_θ for macromolecules is usually much lower than 1 and depends on size, charge, and shape of the macromolecule. It should also be noted that proximal tubule albumin reabsorption contributes to the low urinary albumin concentration. Hence, _g_θ_Alb_ can only be determined from urinary albumin levels if the modification of urine by proximal tubule cells is blocked. Alternatively, the albumin/tracer concentration in Bowman's space or early proximal tubule must be quantified. Functional models of the GCW (20–24), derived from experimental sieving data for infused tracers like ficoll (25–28), dextran (29–31), albumin (32–34) or endogenous circulating proteins (28, 35, 36), suggest that the GCW functions as a composite gel-like mesh with a high density of pores having a radius in the 45–50 Å range, a few large pores with radii of 75–155 Å, and a negatively charged layer at the blood/endothelial interface with a charge density of ~35–45 mEq/L (22). Taking into account these experimentally derived parameters, a mathematical model predicted a _g_θ_Alb_ of 2 × 10^−3^ (a ratio of ultrafiltrate: plasma albumin of 2: 1,000) (22). In fairly close agreement, the best measured estimate of _g_θ_Alb_ obtained by micropuncture in rats was 6.2 × 10^−4^ (0.62: 1,000) (33), and _g_θ_Alb_ derived from radiolabeled albumin tracer studies (34) was 6 × 10^−4^. More recent quantification of _g_θ_Alb_ by intravital two-photon fluorescence microscopy in rats has varied more widely: 0.034 (37), 0.014 (38), 0.002–0.004 (39) and 0.00044 (40). It appears that technical limitations account for some of the higher values by this approach (39, 41). Norden et al. (36) studied humans with the Fanconi syndrome due to Dent's disease, in whom proximal tubule albumin reabsorption is negligible, and found that _g_θ_Alb_ averaged 7.7 × 10^−5^. Similarly, when megalin and cubulin were conditionally deleted in mice (42, 43), _g_θ_Alb_ was estimated at 7.5 × 10^−5^ and 1.7 × 10^−5^, respectively. In such mice, streptozotocin diabetes (43) or superimposed podocin (42) deletion resulted in a significant increase in _g_θ_Alb_. Since proximal tubule uptake of albumin was completely absent in megalin/cubulin deficient mice (42), these data, taken together with those from rats and humans, indicate that ~0.01–0.1% of plasma albumin passes through the GCW into Bowman's space. In normal humans therefore, an estimated 500–5,000 mg of albumin are filtered each day (44). Proximal tubule uptake then reduces excretion to <30 mg/day.

### Size and Charge Selectivity

Mathematical models of sieving data (20, 24, 25, 35, 45, 46) agree that the GCW is best described as a hydrated gel that hinders entry and movement of macromolecules based on size, shape, flexibility and charge (21, 22). Gaps in the gel that allow relatively free filtration of water and small solutes are modeled as abundant small “pores” with a molecular radius cutoff in the 45–50 Å range. The models include a small number of larger “pores” to account for the transit of a small fraction of large macromolecules. It turns out that the shape and flexibility of macromolecules influence movement through the small, abundant gaps given that large, elongated uncharged carbon nanotubes seem to be filtered relatively freely (47). Recent data furthermore suggest that compression of GCW components against intact podocytes may influence the size of gaps in the gel and therefore the molecular size cutoff (48). Abundant experimental data in animals (25–27, 30, 34, 49–52) and humans (20, 29) and ensuing mathematical models (20, 22, 27, 53–56) have concluded that for molecules like albumin whose size is close to the 45–50 Å radius cutoff, negative charge also impedes movement into and through the gel, compared to the same or similar neutral molecule. Conversely, neutralization of negative charges in the GCW with cationic protamine sulfate (57, 58), hexadimethrine (34, 59) or their removal with neuraminidase/sialidase (60, 61) which strip sialic acid from the GCW, all increase albumin excretion rapidly and reversibly. However, because these interventions also cause structural changes in podocytes and glomerular EC, the cause-effect relationship specifically between the reduction in GCW negative charge density and albuminuria was not proven. Nonetheless, infusion of enzymes to destroy negatively charged glycosaminoglycans (GAGs) also increase the fractional clearance of albumin across the GCW (62, 63), even without changes in EC or podocyte ultrastructure. By contrast, in isolated GBM, no change in albumin permeability was observed when negative charges were neutralized with protamine (64), and the substantial reduction of GBM negative charges due podocyte-specific deletion of agrin ± perlecan (65), or the heparan sulfate glycosyltransferase EXT1 (66) raise albumin excretion only minimally. While a change in _g_θ_Alb_ in the knockout mice could have been masked by proximal tubule albumin reabsorption, the results nevertheless cast some doubt on the possibility that “fixed negative charges” located in the GBM play a major role in charge selectivity.

### Location of the GCW Albumin Barrier

The first detailed transmission electron microscopy (TEM) studies of glomeruli caused Farquhar (67) to rule out the glomerular endothelium as a component of the barrier because its fenestrae, lacking visible proteinaceous diaphragms, seemed simply too large to restrict anything smaller than circulating cells. Hence, the glomerular basement membrane (GBM) (68) and podocyte filtration slit diaphragms were held to be the main barrier to macromolecule filtration, with charge selectivity assigned to the GBM (46, 69, 70). This deduction was strengthened by findings of negatively charged sites within the GBM (71–73), congruent functional studies showing charge selectivity of the GCW (34, 51, 74), and the fact that disorders affecting podocytes or GBM all lead to proteinuria (75).

Nonetheless, the concept that the GBM and podocyte slit diaphragm constitute the main barrier to GCW protein flux cannot be reconciled with the fact that bulk convective transit of macromolecules through wide open glomerular endothelial fenestrae would rapidly clog the filter unless high-capacity mechanisms returned them, intact, to the circulation (76, 77). While podocytes endocytose and degrade albumin and other macromolecules (78), this mechanism does not have the capacity to deal with a daily load of albumin in the 4–7 kg range. Long albumin and immunoglobulin half-lives and a low renal albumin degradation rate (79) are also inconsistent with removal and degradation of massive quantities macromolecules by podocytes. Farquhar (67) suggested that macromolecules pass through the endothelium into the GBM and sub-podocyte space and then are swept into the mesangium. However, bulk transit of plasma proteins through the mesangium back into the circulation has never been demonstrated, and glomerular lymphatics that would be needed to clear them from the mesangium have not been found (80).

It turns out that under physiological conditions, endogenous albumin (81, 82), or infused gold-conjugated albumin (68), actually do NOT penetrate glomerular endothelial fenestrae, leading to the more attractive conclusion that a barrier covering the endothelium and extending into endothelial fenestrae retains all but a small fraction of albumin and other large proteins within the circulation. Indeed, disruption of glomerular EC adherens junctions by EC-specific notch1 activation or VE-cadherin deletion results in glomerular EC glycocalyx damage and significant proteinuria (83), implying that fully differentiated glomerular EC with intact adherens junctions and glycocalyx are critically important components of the GCW barrier. No doubt, as detailed by comprehensive models of GCW permselectivity (48, 84–87), one cannot consider any single GCW component in isolation (88), but the role of the glomerular endothelium in GCW permselectivity, for which data were already accumulating in the 1980's (9, 89) is only now becoming widely accepted (10, 53, 88, 90–95).

## Physical Structure of the Glomerular EC Glycocalyx and Surface Layer

The EC glycocalyx consists of proteoglycans, sialomucins, other glycoproteins and glycolipids, all anchored to EC plasma membrane. Molecules in the EC glycocalyx interact with and extend into the sub-endothelial GBM and into a luminal endothelial surface layer (ESL). The ESL is composed of secreted and circulating molecules that associate reversibly with the luminal EC glycocalyx, forming a hydrated, loose gel-like layer between blood and EC glycocalyx. These delicate EC surface components are destroyed by tissue processing for conventional electron microscopy (EM) due to their hygroscopic nature, and perfusion and oxygenation are required for their stability (81, 96). The luminal EC glycocalyx and the ESL were therefore not appreciated until appropriate techniques for their visualization and quantification were developed.

### Visualization of the EC Glycocalyx

With conventional processing for transmission or scanning EM the glomerular endothelium has the appearance of a sieve, with fenestrae ~60–80 nm (600–800 Å) in diameter accounting for ~30% of the glomerular EC surface area. Glomerular EC fenestrae are plasma membrane-lined, transcellular pores that lack the proteinaceous PV-1-based diaphragms observed in most other fenestrated endothelia (97). The size and density of glomerular EC fenestrae accounts for the enormous hydraulic conductivity of the GCW (98). Any decrease in their density and/or size leads to a reduction in GFR, for instance in experimental models of uranyl nitrate (99) and gentamicin (100)-induced acute renal failure, streptozotocin induced diabetes (101), and in humans with diabetes (102) and preeclampsia (103).

**Figure 1:**
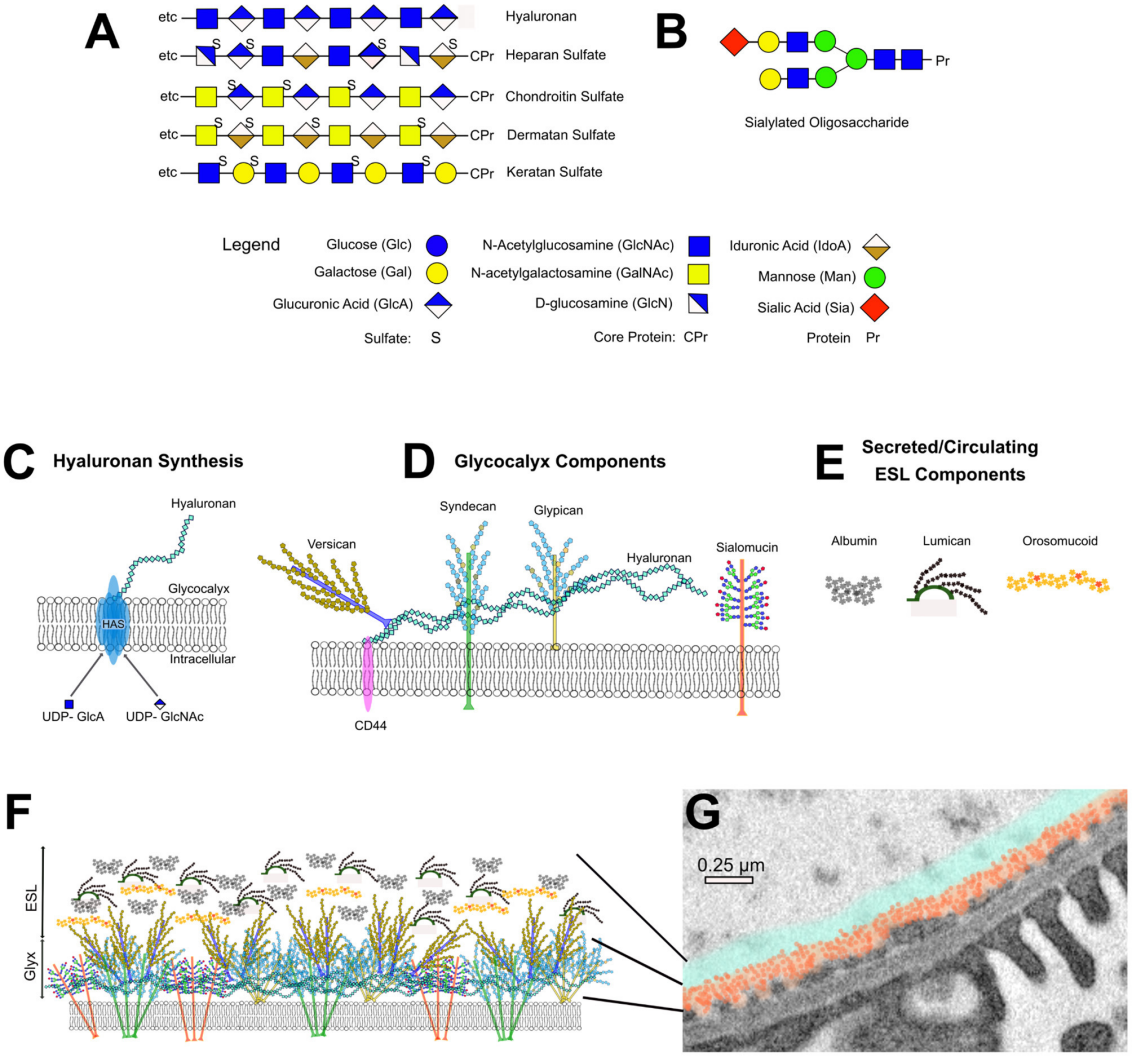
Components of the EC glycocalyx and ESL. **(A)** Structure of glycosaminoglycan chains. **(B)** Example of a branched, sialylated oligosaccharide side-chain. **(C)** Schematic representation of hyaluronan synthase (HAS)-mediated hyaluronan synthesis at the cell membrane. **(D)** Schematic representation of major EC glycocalyx components. **(E)** Schematic representation of major ESL components. **(F)** Artistic representation of the dense, bundled glycocalyx component. **(G)** Transmission EM image of a glomerular capillary wall with superimposed artistic representation of the glycocalyx (orange) and ESL (blue) thickness.

The radius of glomerular EC fenestrae is much larger than the effective radius of circulating macromolecules that are not filtered, for instance orosomucoid (29 Å), albumin (36 Å), Transferrin (43 Å), IgG (55 Å), α2-macroglobulin (90 Å) and fibrinogen (108 Å) (35, 104), so they were initially assumed to allow their free convective movement into the GBM. Yet, studies in non-glomerular capillaries had suggested that EC fenestrae are impermeant to macromolecules (104), and Luft (105) found that EC do not present a “naked” surface to circulating plasma, given that perfused electron-dense ruthenium red accumulated on the EC luminal surface revealing an anionic coat. Avashi and Koshy (106) perfused kidneys with ferritin, a multimer ~120 Å nm in diameter, so much smaller than glomerular EC fenestrae. Cationic ferritin densely decorated the glomerular EC surface and the core of fenestrae and did not penetrate into the GBM. Anionic ferritin was completely excluded from the EC surface and the GBM, indicating that the EC coat excludes negatively charged macromolecules. Furthermore, adhesion of cationic ferritin was removed by neuraminidase and reduced by heparinase and hyaluronidase without change in EC or podocyte ultrastructure. The authors concluded that “*glomerular endothelial fenestrae are not empty holes” but “are occupied by an anionic matrix that is visualized only following the binding of an electron-dense tracer. In this respect the matrix in the fenestrae is similar to the glycocalyx at the external surface of cells which also remains invisible in unstained preparations*” (106). Rostgaard and Qvortrup (96) extended these observations using oxygen-carrying perfusion fixation and tannic acid/uranyl acetate staining. They observed “sieve plugs” in fenestrae of intestinal and peritubular capillary EC, and a similarly stained layer covering the EC. But in glomerular EC the same procedure revealed only a delicate ~300 nm thick surface coat (96). Hjalmarsson et al. (107) reported a colloidal lanthanum labeled ~60 nm thick EC glycocalyx that was revealed in oxygen-carrying perfusion fixed, tannic acid-stained tissue. They observed a thicker ~200 nm coat ascribed to glycocalyx plus ESL. In their study, cupromeronic blue stained tissue showed a semi-ordered proteoglycan network within the fenestrae (107). In glomerular EC, Hegermann (108) recently visualized an amorphous 200–300 nm thick layer with alcian blue. With cationic colloidal thorium they observed an electron-dense layer that filled the fenestrae, extended from the EC surface by 50 to 300 nm and was organized into bundles that were about 50 nm wide at the EC surface, with sub-organization into wider and wider bundles as they moved away from the surface. They concluded that the glycocalyx proper represents bundles of proteoglycans that are anchored to the EC plasma membrane and extend vertically from the cells toward the capillary lumen (Figure 1). These findings are consistent with those by Squire et al. (109) in non-glomerular EC, who reported vertically organized bundles extending from the EC surface, intertwined with horizontal strands forming a lattice with gaps that could account for size-selectivity. Indeed, Fan et al. (110) were able to visualize hyaluronan (HA) and heparan sulfate (HS) at the single molecule level in cultured EC, using stochastic optical reconstruction microscopy (STORM), i.e. a super-resolution imaging technique with a resolution of 20 × 50 nm. They reported that HS bundles extend vertically from the EC surface and are intertwined with horizontally arranged, long HA strands to form an organized lattice-like network on the EC surface (110, 111).

### Defining the Height of the ESL

It could still be argued that tissue processing and deposition of electron-dense material for transmission EM could produce artifact that might overestimate the dimensions of the EC glycocalyx and/or might remove the ESL. To assess the thickness of the EC glycocalyx/ESL the zone of exclusion for RBCs or fluorescently labeled tracers above the EC plasma membrane is therefore commonly determined. For instance, in hamster cremasteric muscle capillaries, the exclusion zone for dextran 70 and RBCs was found to be 400–500 nm (112), and was significantly reduced by hyaluronidase, and partially reconstituted hyaluronan or chondroitin sulfate infusion (113). In renal glomerular capillaries, the zone of exclusion for infused intralipid droplets was ~200 nm and was also significantly reduced by enzymes that cleave glycosaminoglycans (114) and by elution of ESL components with hypertonic NaCl (115). Evaluation of the EC glycocalyx/ESL thickness is now a commonly used technique in human clinical research (116) and has helped define changes in the height of the glycocalyx/ESL in disease.

Hence, all EC surfaces are covered by an organized glycocalyx and an associated ESL. These form an anionic surface that results in repulsion of anionic macromolecules as well as the anionic glycocalyx of circulating cells. The anionic EC glycocalyx extends into the fenestrae, forming a semi-permeable matrix that allows rapid filtration of water and small solutes, but not macromolecules. The lattice-like arrangement of the EC coat furthermore suggests that it participates in size-selectivity of the GCW. It is already well-established that immune-activation of EC changes its glycocalyx, breaching the normal glycocalyx/ESL (116), allowing EC interactions with circulating cells and platelets and facilitating thrombosis (117). Under those conditions it is therefore expected that permselectivity is also reduced.

## Molecular Components of the EC Glycocalyx

The EC glycocalyx is composed membrane-anchored proteoglycans and sialomucins that in conjunction with secreted, hyalectin-bound hyaluronan (HA) form an organized, extremely hydrated lattice-like gel. Many membrane-anchored glycoproteins embedded in the glycocalyx serve as receptors for cytokines, growth factors and as counter-receptors for circulating cells (117). The ESL, on the other hand is a concentrated layer of circulating and EC-secreted proteins, glycoproteins, small proteoglycans and other macromolecules, that is in dynamic equilibrium with the circulation (118).

### Glycosaminoglycans

The structure and function of the EC glycocalyx depends critically on its glycosaminoglycan (GAG) composition. GAGs are long, unbranched polymers of repeating disaccharides, each consisting of an amino sugar (N-acetylglucosamine or N-acetylgalactosamine) and either a galactose or uronic acid sugar (Figure 1A). Due to their high hydroxyl and sulfate content, GAGs are negatively charged; they bind large amounts of water, critical for their viscoelastic properties; they repel negatively charged molecules like albumin and they often serve as co-receptors for growth factors and cytokines. In the luminal EC glycocalyx GAGs confer anti-coagulant properties, they repel circulating cells, and they impart the charge barrier to the endothelium. Heparan sulfate (HS), chondroitin sulfate (CS)/dermatan sulfate (DS) and keratan sulfate (KS) GAGs are all assembled on core proteins of distinct proteoglycans. Hyaluronic acid (HA; aka hyaluronan) (119) is the only GAG synthesized outside the Golgi as a stand-alone polysaccharide composed of non-sulfated N-acetylglucosamine/glucuronic acid disaccharide repeats. HS (120), the most abundant GAG in the EC glycocalyx, consists of N-acetylglucosamine/uronic acid repeats, CS GAGs (121) consist of N-acetylgalactosamine/glucuronic acid and KS (122). GAGs are composed of N-acetylglucosamine/galactose disaccharide repeats (Figure 1A). Addition of GAG chains to proteoglycans in the Golgi is initiated by specific glucuronyl transferases that covalently couple a bridging tetrasaccharide through O-linkage on serine or threonine or N-linkage on asparagine, followed by elongation of the polysaccharide chain and subsequent position-specific modifications through de-acetylation/sulfation and epimerization. For instance, within the initial HS polymer, epimerization converts some of the glucuronic acids to iduronic acid (123) and de-acetylation converts some of the N-acetylglucosamine to glucosamine. Similarly, CS is converted to DS through epimerization of glucuronic to iduronic acid. For proteoglycans, structural and functional diversity is therefore not only due to their different protein cores, but also the GAG type, the number and length of their GAGs, as well as position-specific epimerization and sulfation. Since enzymes involved in GAG-core protein attachment, chain elongation and modification serve multiple proteoglycans, any mutations or deletion in the many enzymes that carry out these functions tend to have different, and often more severe phenotypes than mutations or deletion of any one proteoglycan core protein (124).

Cleavage by enzymes that are GAG-specific, namely hyaluronidases, heparinase and chondroitinase have been used extensively to define the functional role of GAGs in the GCW and EC glycocalyx, and shedding of HA and EC cell-surface proteoglycans (125) into the circulation due to endogenous enzymes is used as an indicator of glycocalyx damage (126, 127).

### Hyaluronic Acid/Hyaluronan

In vertebrates HA is produced by HA synthases encoded by three distinct genes (HAS1-3). The HA synthases are integral plasma membrane proteins with multiple membrane-spanning domains (Figure 1C). The catalytic site within their hydrophilic core acts as a polymerase, converting soluble intracellular UDP-GLcNAc and UDP-GLcA to polymeric HA, simultaneously extruding the growing polymer it into the extracellular space (128). The length of the HA polymer varies from about 1,000 to 10,000 kDa. The rate of HA synthesis depends on the availability of substrate sugars (129), and conversely, high rates of HA synthesis are associated with a shift of cellular metabolism to glycolysis (130–132). HA binds to cell-surface receptors CD44 (133), RHAMM (receptor hyaluronan mediated motility) (134) and the lymphatic EC receptor LYVE-1. The cytoplasmic domain of CD44, a single pass membrane-spanning receptor, is coupled to cortical actin by ERM (ezrin radixin moesin) proteins (135). The interaction of CD44 with HA enhances EC barrier function (136) and is necessary for transmission of shear force signals that cause Rac1-dependent EC re-orientation (137), enhanced nitric oxide synthesis (138) and increased HAS2 expression (129, 139). In keeping with luminal HA/CD44 interactions, HA loss from the glycocalyx profoundly reduces shear-force induced NO synthesis (140).

HA turnover is rapid and regulated, in part, through HA degradation by hyal-1 and−2 (Hyaluronidase-1 and−2) and by Cemip-1 and−2 (Cell Migration-Inducing hyaluronidase-1 and−2; the latter also known as transmembrane protein 2/TMEM2) (131, 141). Hyal-2 is a GPI-anchored plasma membrane-associated enzyme that cleaves CD44-bound HA. The fragments are then internalized by the GAG scavenger receptor HARE (142). The cemid1 (143) and−2 (144) hyaluronidases are single-pass plasma membrane-spanning proteins that degrade extracellular HA into small, bioactive extracellular fragments (oHA). These oligosaccharides modify VEGF signaling in EC (144).

HA polymers are hydrated with 15 H_2_O molecules per disaccharide unit (145). At the apical/luminal EC surface HA interweaves with other components of the glycocalyx/ESL (110, 134) and shear stress (129, 146) and inflammatory stimuli (147) augment HA accumulation in the EC glycocalyx. HA binds proteoglycans in the hyalectin family (see below) forming large, patterned aggregates. A HA-versican lattice may in fact account, at least in part, for the semi-ordered appearance of the apical EC glycocalyx (108, 109), and perhaps also contribute to size-selectivity of the GCW.

In systemic microvessels, destruction of HA by hyaluronidase markedly reduces the height of the EC glycocalyx and its macromolecular barrier function (113). Similarly, in glomerular capillaries the height of the EC glycocalyx is reduced by hyaluronidase infusion along with an increase in the fractional excretion of albumin (114). EC-specific, conditional HAS2 deletion in mice reduced glomerular EC HA and cationic ferritin labeling, along with progressive proteinuria, glomerular EC ultrastructure changes and capillary involution (148). Conversely, in the mouse streptozotocin model of diabetes deletion of the HYAL1 gene reduced hyaluronidase activity, preserved the EC glycocalyx and was associated with less glomerular barrier disruption than in wild-type mice (149). These findings are consistent with those in diabetic patients, where higher levels of circulating HA and hyaluronidase were found to be associated with the development of microalbuminuria (150, 151), and where endothelial glycocalyx disruption was associated with a substantial reduction in glomerular endothelial HA content (148).

### Endothelial Proteoglycans

Among many glycosylated proteins, proteoglycans are distinguished by their very long, unbranched, sulfated GAG sidechains usually accounting for at least 60% of their molecular mass, the exception being perlecan, where the GAG chains are a minor component (see below). Classification of distinct proteoglycans is based on the structure of their protein core, the type and number of associated GAG chains, and their molecular interaction profile. Some proteoglycans are integral membrane-spanning proteins, some are covalently bound to the outer leaflet of the plasma membrane by GPI (glycosylphosphatidylinositol) anchors, and others are secreted. The principal structural glycocalyx proteoglycans in EC are membrane-spanning syndecans, GPI-anchored glypicans and secreted perlecan and versican. Other small, secreted proteoglycans are produced by EC and participate in defining the dynamic EC phenotype. In addition to their GAG chains, proteoglycans can also be modified by branched oligosaccharide side chains, some terminated by sialic acid. There currently is a paucity of data on potential effects of such modifications on the properties of the glycocalyx/ESL. Thus, future studies are needed to better understand their impact on glomerular permselectivity.

#### Syndecans

Syndecans 1–4 are ubiquitous single-pass type I membrane-spanning proteoglycans, with core proteins in the 20–45 kDa range. The extracellular domains of syndecan-1 and−3 are decorated by HS and CS GAGs, while syndecans-2 and−4 contain only HS GAGs. Their GAG-rich extracellular domains interact with many growth factors, cytokines and extracellular matrix proteins transmitting signals *via* their cytoplasmic domains down several intracellular pathways (152–155). In EC, syndecans act as co-receptors promoting angiogenesis (156–158) and the EC response to inflammation (158–160). At the basal surface of angiogenic EC, syndecan-1 is part of integrin/focal adhesion complex (157, 161) that promotes angiogenesis, and both syndecan-4 (162) and syndecan-1 (163) participate in the EC remodeling response to shear stress. All syndecans are expressed in cultured glomerular EC where they are part of the luminal/apical EC glycocalyx (127, 159, 164). In Zebrafish *in vivo*, syndecan-3 is the main syndecan in glomerular EC (165).

The EC response to inflammation is characterized by upregulation of syndecan expression (158, 160) and shedding of syndecans from the glycocalyx into the circulation (125, 166), resulting in less syndecan in the ESL (167). The increased mRNA expression levels may be compensatory of the increased shedding of proteins (168). Syndecan shedding is observed in response to thrombin activation (166, 169), hypoxia, ischemia-reperfusion injury (170) and in preeclampsia (171). In glomerular EC, syndecan-4 shedding in response to IL-1β activation and was mediated by matrix metalloproteinase-9 (127). Protease-dependent syndecan shedding (126, 127, 160, 172, 173) produces bioactive soluble syndecan fragments generally inhibiting the inflammatory response (160). Syndecan shedding is now widely recognized as a biomarker of EC glycocalyx disruption (174) and is associated with a reduced glycocalyx thickness and a reduced barrier function resulting in edema formation and albuminuria (175). While cleavage of HS and CS GAGs reduces the size and barrier of the glycocalyx in glomerular and non-glomerular EC, endothelial-specific deletion of syndecan 1 alone only reduced the height of the glycocalyx but did not change its barrier function (176). In cultured EC, sphingosine-1-phosphate (S1P) rescued shedding of syndecan-1 and glycocalyx GAGs due to plasma protein depletion (177). Since S1P is presented to EC by albumin, this was taken to indicate that the effect of serum proteins on glycocalyx integrity may be mediated by S1P (178, 179). Substantial syndecan shedding along with thinning of the EC glycocalyx has also been reported in patients with CKD where it correlates with markers of EC dysfunction (180). Hence, the syndecans along with their HS GAGs are major contributors to the EC glycocalyx thickness. Their shedding signals EC glycocalyx dysfunction along with a reduction in the EC glycocalyx barrier to protein filtration.

#### Glypicans

Glypicans (124, 181) are proteoglycans composed of 60–70 kDa core proteins with heparan sulfate GAG side chains. The C-termini of glypicans are attached to the plasma membrane through GPI anchors. There are 6 glypican genes (GPC1-6), among these, glypican-1 is predominant in EC (182). Glypicans enhance fibroblast growth factor (FGF) (183), and VGEF-dependent (182) cell proliferation, in turn stimulating angiogenesis. Due to its GPI anchor, glypican-1 localizes to lipid microdomains often referred to as rafts, and clusters in response to shear stress in EC caveolae (184), where it activates NO synthesis in response to traction forces (163, 185, 186). In glypican-deficient mice, NO synthesis is markedly reduced, in keeping with a major role of EC glypican-1 in signaling NO synthesis (187). Conversely, the HS GAGs of glypican-1 undergo non-enzymatic cleavage from their core protein by NO (124), resulting in glypican-1 endocytosis and recycling (188). Reduced NO synthesis, a hallmark of EC dysfunction in inflammatory diseases and under conditions of increased oxidative stress, has therefore been attributed to reduced EC glycocalyx glypican-1 function (174).

#### Versican

Versican and aggrecan, both abundant in the vasculature, belong to the family of hyalectins (189), large, secreted CS-containing proteoglycans that bind hyaluronan with high affinity forming aggregates with substantial viscoelastic strength (190). Like other proteoglycans, hyalectin GAGs bind growth factors and cytokines, regulating their interaction with cell-surface receptors, and their cleavage by proteases releases bioactive fragments (189). In EC, synthesis of an HA-binding CS proteoglycan by EC was first demonstrated by Morita et al. (191) and versican was subsequently shown to be produced by EC (192), including glomerular EC (164). Aggrecan is synthesized by vascular smooth muscle cells and myofibroblasts (193) but evidence for its synthesis by EC is lacking so far. While versican is part of the subendothelial matrix where it binds the matrix protein fibulin (194), it also localizes to the apical/luminal EC surface where it binds hyaluronan which, in turn, attaches to cell-surface CD44 (189). Co-localization of CS GAGs and hyaluronan on the apical surface of immortalized glomerular EC in culture has been documented and removal of CS reduced the transendothelial resistance and increased apical to basal albumin flux (195) indicating a role for CS containing proteoglycans in the EC barrier function. In zebrafish, versican was observed in glomerular EC and podocytes, and its knockdown reduced the barrier function of the GCW (165). Versican synthesis by cultured glomerular EC is inhibited by puromycin aminonucleoside (164). Adriamycin *in vivo* similarly reduced glomerular versican expression along with a profound loss of glomerular EC glycocalyx/ESL thickness and an increase in the sieving coefficient for albumin due to a charge defect (196). A similar charge defect was associated with reduced glomerular versican expression in diabetic mice (197). In aggregate, these studies indicate that versican is part of the glycocalyx that surrounds EC, and that its GAGs participate in glomerular charge selectivity.

#### Perlecan

Perlecan is a massive proteoglycan whose protein core alone has a molecular mass of ~470 kDa and is composed of 5 distinct functional domains (152, 198). Three GAG chains, which can be HS, CS or KS, decorate the N-terminal perlecan domain, each contributing another ~40 kDa to the overall molecular mass. The C-terminus of perlecan interacts with transmembrane integrins. Produced by all EC (164, 199), perlecan carries only HS GAGs in EC, and is secreted into the subendothelial matrix and the EC apical/luminal surface layer (199, 200). A host of molecular interactions specific for each of the 5 perlecan domains have been described, and proteolytic cleavage of perlecan produces bioactive fragments (152, 198, 199). Relevant for EC is the pro-angiogenic action of intact perlecan and the anti-angiogenic function of endorepellin, the cleaved, soluble perlecan V domain that inhibits VEGFR2 in EC (199). Perlecan functions as a mechanosensor at the surface of chondrocytes where it transmits shear stress signals produced by compression-induced fluid flow in cartilage canaliculi. A similar function as a shear stress sensor has been proposed for EC (152, 198), though is no proven so far. Perlecan deletion in mice is lethal, but the knock-out mice are viable when perlecan is selectively rescued in chondrocytes (201). In that model, EC perlecan is required for the appropriate formation of EC cel-cell junctions and pericyte recruitment by brain microvessels (201). In mice carrying a perlecan mutation that precludes attachment of its GAG chains, no abnormalities in glomerular structure or function were detected, and the macromolecular GCW barrier function remained intact (65). So, while perlecan is a major proteoglycan produced by EC, its GAG components do not seem to confer charge-selective properties to the GCW, and absence of perlecan GAGs do not impair glomerular EC ultrastructure. Nonetheless, perlecan shedding from the glycocalyx has been observed under conditions of EC dysfunction. For instance, in patients with severe preeclampsia circulating perlecan levels are significantly higher than in normal pregnant women (202).

#### Small Leucine-Rich Proteoglycan Family

The (SLRP) family (203) includes decorin, biglycan and lumican all produced by EC, including glomerular EC in culture (164) and *in vivo* (204, 205). These SLRPs are characterized by a small core protein (~ 40 kDa) with few CS/DS or KS GAG chains (206). They are secreted into the subendothelial matrix where they interact directly with collagen, aiding in the structural matrix organization and EC adhesion and migration (207). Lumican is found in a high-salt eluate of renal vessels (115, 208), suggesting that it is a major component of the ESL. The SLRPs interact with, and regulate the function of TGF-β and its family members and other growth factors (203). Most recently decorin was shown to activate the autophagy pathway in EC (209). Decorin, biglycan and lumican null mice have been created, but so far roles in defining glomerular EC ultrastructure, thickness of the glomerular EC coat, or glomerular permselectivity have not been reported.

#### Endothelial Specific Molecule-1

Endothelial specific molecule-1 (ESM1, aka endocan) is a small, secreted EC-specific CS/DS proteoglycan (210) induced by TNF-α and IL-1β. It interacts with integrins and growth factors and is involved in regulating angiogenesis. Its circulating levels increase and correlate with microalbuminuria in patients with hypertension (211).

#### Serglycin

Serglycin is a small proteoglycan expressed by EC, and hematopoietic cells (212, 213) whose name refers to a serine/glycine repeat domain that supports attachment of several GAGs through O-linked glycation on Ser residues. At baseline, serglycin is sequestered in intracellular granules and participates in granule mobilization in response to inflammatory stimuli. In activated EC, serglycin promotes cell-surface localization of chemokine receptors (213, 214).

### Endothelial Sialomucins

Sialomucins in the EC glycocalyx are integral plasma membrane glycoproteins each with a single membrane-spanning domain, a large extracellular “mucin” domain and a cytoplasmic domain that interacts with cortical actin *via* ERM (ezrin radixin moesin) proteins. Mucin domains are ser/thr/pro-rich regions densely decorated by O-glycans initiated by core 1 β1,3 galatosyltransferase (215) and containing terminal sialic acids. Silomucins largely accounting for the high sialic acid content of the EC glycocalyx. Several sialomucins, including podocalyxin (216, 217), endoglycan (218) (aka podocalyxin 2), CD34 (219), and endomucin (220, 221) are expressed by EC, while podoplanin is restricted to lymphatic EC (215). Sialomucins are sorted to the apical/luminal surface (216, 222) of EC where they play a repulsive role during embryonic vascular lumen formation (222, 223) and they repel circulating cells by virtue of their negative charge (219). EC sialomucins (219) play a role in hematopoietic precursor trafficking (219) and as counter-receptors for L-selectin, though this latter function requires modification of the O-glycan by carbohydrate 6-*O*-sulfotransferase restricted to high endothelial venules (224). The potential role of sialomucins the glomerular EC barrier to macromolecule flux has only been studied indirectly, through infusion of neuraminidase (60, 61, 95, 106, 225–227), which removes sialic acid from the GCW and consistently results in albuminuria. However, since podocytes also express the sialomucins podocalyxin and podoplanin, it is possible that the neuraminidase-induced GCW barrier results from stripping of sialic acid from both, EC and podocyte sialomucins. Even so, in cultured EC, podocalyxin knock-down markedly reduces the trans-endothelial resistance. EC-specific podocalyxin deletion in mice alters EC structure and reduces the EC barrier function in lung and brain in the presence of pro-inflammatory stimuli (228–230). Global podocalxyin deletion in mice is lethal due to a major podocyte defect, though in these mice glomerular EC are also thickened and lack fenestrae (231). Conditional deletion of the core 1 β1,3 galatosyltransferase, critical for sialylation of all sialomucins, results in marked albuminuria (232). Finally, in children with streptococcus pneumoniae associated HUS, neuraminidase-mediated removal of sialic acid from sialoglycoproteins in the EC glycocalyx likely plays a significant role in triggering intravascular coagulation, hemolysis, and acute renal failure accompanied by proteinuria (233).

## Circulating Proteins in the Endothelial Surface Layer

The ESL refers to a layer of macromolecules that merges with glycocalyx GAGs substantially increasing the separation of freely flowing plasma from the EC surfce (118). The height of glycocalyx with ESL is ~250 nm in glomerular capillaries (114), and up to 500–1,000 nm in systemic vessels (113). The loosely bound macromolecules of the ESL, some secreted by EC, others derived from circulating blood (Figure 1), are in dynamic equilibrium with flowing plasma and are concentrated in the zone above the glycocalyx due to the sieving effect.

The precise composition of the ESL is not known, though it contains albumin, orosomucoid, lipoproteins, lipases, complement components, and small proteoglycans secreted by EC, like lumican (115). Removal of GAGs and terminal sialic acids disrupts the interactions of ESL components with the glycocalyx proper, causing glycocalyx collapse and a reduction in the zone of exclusion.

### Albumin

Produced by the liver at a rate of ~10 g/day, albumin is the most abundant circulating protein, with normal plasma concentrations of 35–50 g/L and a half-life of 19–20 days. Encoded by a single gene, human albumin is secreted as a monomeric non-glycosylated polypeptide consisting of 585 amino acids (MW ~66.5). A relatively high content of acidic amino acids and fatty acid binding result in an estimated isoelectric point of 4.7–5.8 (234). Hence, in physiologic solutions albumin is negatively charged. Structural analyses (235–238) show that albumin is not a simple sphere, but that it consists of 3 major domains, each containing subdomains, with 17 intramolecular disulfide bonds contributing to 3D folding. Normally, albumin assumes a heart-shaped triangular structure (237) with a hydrodynamic radius of 36.2 Å, though it can assume other conformations depending on pH, including an expanded cigar-like shape with a hydrodynamic radius of 61.5 Å (238). Were it not for its negative charge, the structure of albumin and its ability to take on different conformations suggest it could penetrate a meshwork with mean pore radii in the range of 40–60 Å, like the glomerular capillary wall (20). The albumin monomer contains hydrophobic pockets that bind many lipophilic substances, among them endogenous fatty acids, steroid hormones, thyroid hormone, bilirubin, vitamins, and phytochemicals. Its binding affinity for many drugs and its potential as drug carrier have been extensively investigated. Non-enzymatic glycation of albumin results in conformational changes that alter its interaction with endogenous substances and drugs, increase its half-life and reduce formation of albumin aggregates (38, 239).

While albumin flux across the endothelial glycocalyx and ESL is highly restricted (81), albumin also associates with the ESL and alters the endothelial barrier function. *In vitro* NMR studies show interactions between albumin and hyaluronan resulting in albumin/hyaluronan complexes that hinder the mobility of albumin in solution (240). Albumin binds to immobilized artificial glycocalyx composed of hyaluronan, heparan sulfate and chondroitin sulfate GAGS though its binding affinity is low (241). In cultured EC, albumin similarly associates with the EC cell surface in a reversible fashion (242), and in perfused frog mesenteric microvessels (243) endogenous albumin was observed in a ~200 nm thick layer covering the EC surface (243). Likewise, albumin associates with lung EC glycocalyx; Lowering perfusate plasma protein/albumin content significantly increased penetration of endogenous, negatively charged ferritin into the vessel wall (244). Similarly, in isolated dog glomeruli, lowering perfusate albumin concentrations raised GFR not only due to a reduction in the colloid osmotic pressure, but also due to an increase in the hydraulic conductivity of the glomerular capillary wall (245). A similar effect of albumin on the hydraulic conductivity was also reported for non-glomerular vessels in frog (225) and rabbit (246). Finally, in the analbuminemic Nagase rats, the negative charge density of the glomerular EC coat was reduced, with enhanced penetration by macromolecules in the 60–90 kDa range both corrected by albumin infusion (247). In tracer studies, enhanced flux of glycated albumin across the EC layer has been reported (248), though by two-photon microscopy its GCW sieving coefficient was not different than that of native albumin (38). Instead, reduced uptake of glycated albumin by the neonatal Fc receptors (FcRn) in proximal tubule cells enhanced its renal excretion (38). Thus, *in vitro* and *in vivo* studies all indicate that albumin associates with the EC coat, reducing the filtration coefficient and the trans-endothelial flux of macromolecules (179). The relatively low affinity of albumin for glycocalyx/ESL components furthermore suggests that bound albumin is constantly exchanged with circulating albumin. Given that albumin binds the bioactive lipid S1P (178, 249), and that S1P protects the EC glycocalyx (250), it is likely that albumin not only changes the function of the endothelial glycocalyx/ESL through physical binding, but that it also delivers mediators to the EC that alter glycocalyx/ESL synthesis and degradation.

### Orosomucoid

In humans, orosomucoids are produced by two distinct genes, ORM1 and 2. Orosomucoids are sialylated, negatively charged circulating glycoproteins produced mainly by the liver (251) but also by EC (252). Basal plasma concentrations are in the range of 1 g/L. Orosomucoid synthesis is strongly induced by inflammatory stimuli, like lysopolysaccharide (LPS), and interleukins-1 and−6; they are therefore considered to be acute phase reactants (251). Orosomucoid core proteins (~21.5 kDa) undergo complex and variable glycosylation prior to secretion, increasing their molecular mass to ~ 44 kDa, and resulting in a high sialic acid content. Orosomucoid glycosylation is modified in response to acute inflammatory stimuli, increasing the density of sialyl-Lewis × epitopes (sLe^x^) that can interact with EC surface P- and E-selectins (253, 254). In cultured EC, orosomucoid 1 binds both high affinity, relatively low capacity cell surface receptors, and lower affinity, extremely high capacity binding sites (255). The former likely represent EC P- and L-selectin binding, the latter association with the ESL, increasing the ESL negative charge density (256). Pertinent to this discussion, orosomucoid reduces the flux of albumin across rat hindlimb microvessels (257), and lactalbumin flux across frog mesenteric vessels (258) and the blood brain barrier (259). In the kidney, perfusate containing orosomucoid reduces the fractional clearance of albumin (32, 260), and administration of of orosomucoid protects rats from puromycin aminonucleoside—induced albuminuria and GFR loss (261). Hence, orosomucoid, which is not filtered but associates with the surface of EC, reduces albumin flux across EC, by increasing the ESL negative charge density. Orosomucoid-dependent modulation of inflammatory cell recruitment and EC transmigration may also contribute to the renal response to injury. For instance, urinary excretion of orosomucoid increases in patients with type 2 diabetes and may be a biomarker for EC dysfunction due to low-grade inflammation (262). In triple (ORM1-3) knockout mice (unlike humans, mice have 3 ORM genes), enhanced inflammation and a greater susceptibility to renal fibrosis in the unilateral ureteral obstruction (263) and acute ischemia-reperfusion (264) models have been reported. At this time, quantitative glomerular permselectivity studies in ORM deficient mice are lacking.

## Disruption of the EC Macromolecular Barrier in Disease

A thorough understanding of the GCW barrier not only requires knowledge of its composition, still incomplete, but also its dynamic regulation. The complete EC barrier consists not only of the glycocalyx/ESL covering the EC surface and filling the fenestrae, but also cell-cell junctions, and the subendothelial glycocalyx/matrix. Even at equilibrium, all constituents of the EC glycocalyx and ESL are continually turning over through tightly regulated mechanisms. They are subject to, and participate in responses to shear and compression forces, to soluble mediators and to signals from podocytes (265–267). Mechanisms disrupting the barrier can range from EC dysfunction observed in the metabolic syndrome and cardiovascular disease, to EC de-differentiation upon withdrawal of critical stimuli like VEGF, observed in preeclampsia, to EC activation by inflammatory stimuli in HUS, TTP and sepsis, all the way to destruction of the EC in some forms of glomerulonephritis and vasculitis.

### Microalbuminuria Reflects Generalized EC Dysfunction

EC dysfunction, characterized by diminished flow-mediated vasodilation due to reduced endothelial NO production, signals generalized EC abnormalities in patients with cardiovascular disease, the metabolic syndrome, diabetes and chronic kidney disease (10, 174, 265, 268). Microalbuminuria is strongly associated with EC dysfunction (269), predicts cardiovascular morbidity (19) and is one of the earliest indicators of generalized, chronic EC injury (9). Note again that microalbuminuria tends to underestimate the GCW defect, due to proximal tubule reabsorption of albumin (79). EC glycocalyx disruption with a substantial reduction in glomerular EC HA content has been documented in patients with diabetic nephropathy (148). In generalized vascular disease, microalbuminuria is associated with a reduction in EC glycocalyx/ESL height and increase in circulating EC glycocalyx components, including hyaluronan (149, 270) and proteoglycans (126, 150, 151, 174). As EC glycocalyx glypican-1 is required to elicit shear-induced NO synthesis (184, 186), it seems likely that glycocalyx degradation is, in fact, the proximate cause of reduced flow-dependent NO synthesis in generalized EC dysfunction. In experimental diabetes, endomucin restored the EC glycocalyx (221, 271), and in human diabetic patients partial restoration of the EC glycocalyx with sulodexide, an orally administered mixture of GAGs, not only lowers blood pressure, but also reduces albuminuria and other diabetic complications (272–276). Hence, microalbuminuria reflects the endothelial barrier defect that accompanies glycocalyx disruption and EC dysfunction in cardiovascular disease, diabetes and chronic kidney disease. The use of GAGs to enhance EC glycocalyx function could well develop into new therapeutic approach. It is important to note that the massive increase in cardiovascular morbidity of dialysis patients is, at least in part, due to chronic EC glycocalyx/ESL dysfunction (277, 278).

### Albuminuria Reflects Glomerular Endothelial Barrier Dysfunction in Preeclampsia

Preeclampsia affects 3–5% of all pregnant women and is associated with substantial risk to baby and mother. Albuminuria and hypertension are the earliest manifestations of preeclampsia. Marked glomerular EC swelling along with loss of glomerular EC fenestrae, also referred to as “glomerular endotheliosis” has long been recognized as the key glomerular abnormality in preeclampsia (279–281). EC abnormalities in preeclampsia, are not restricted to the glomerular endothelium, often involving the choroid plexus as well, and preeclampsia can progress to the full-blown thrombotic microangiopathy of pregnancy (282), the HELLP (hemolysis, elevated liver function tests, low platelets) syndrome. Even so, proteinuria is the main indicator of EC dysfunction in these patients. Glomerular EC differentiation and fenestration depend critically on podocyte-derived VEGF (283) and endotheliosis lesions are observed in mice with podocyte-specific VEGF haploinsufficiency (284). Also, bone morphogenetic protein-9 (BMP-9) signaling *via* the endothelial-specific ALK-1/endoglin receptor complex signals EC differentiation (285, 286). In patients with preeclampsia, placenta-derived, circulating soluble VEGF receptor and soluble endoglin inhibit VEGF- and BMP-9 signaling pathways leading to glomerular EC de-differentiation (3, 287, 288). Along with the ultrastructural EC changes, reduced EC glycocalyx/ESL height and shedding of glycocalyx components into the circulation have been documented in preeclampsia (171, 174, 202, 289). The use of VEGF inhibitors to reduce tumor angiogenesis (290) and macular degeneration (5) can evoke a similar syndrome of albuminuria, sometimes in the nephrotic range, and hypertension. It turns out that the human diacylglycerol kinase epsilon (DGKE) mutation (291), a cause of the hemolytic uremic syndrome, also reflects inhibition of VEGF signaling and consequent de-differentiation of glomerular EC (292). Hence, albuminuria in preeclampsia, and in patients treated with VEGF inhibitors, reflects EC de-differentiation resulting in a breach of the normal glomerular EC barrier to macromolecules.

### The Thrombotic Microangiopathies

Characterized by a vicious cycle of intracapillary thrombus formation, platelet consumption and microangiopathic hemolytic anemia, the thrombotic microangiopathies all involve EC activation (293), whether by Shiga toxin (294), COVID-19 (295, 296), thrombin, complement components (297) and/or inflammatory cytokines (296). Normally, endogenous inhibitors prevent activation of the coagulation and complement cascades at the EC surface and soluble EC-derived mediators like NO and prostacyclin block platelet activation. As part of the luminal EC glycocalyx, integral membrane-spanning thrombomodulin binds and inhibits thrombin, and stimulates protein C, which actively cleaves components of the coagulation cascade (298), by binding to the EC protein C receptor. Tissue factor pathway inhibitor (TFPI) (299) and complement factor H (CFH) (300) both bind HS GAGs in the EC glycocalyx/ESL, inhibiting local thrombin and complement activation, respectively. EC activation also results in reduced NO and prostacyclin production, de novo expression of membrane-anchored tissue factor, release of TFPI and CFH from the EC surface by heparinases (300), and mobilization of P-selectin and von Willebrand Factor (vWF) to capture platelets (301). It follows that even minor causes of EC activation, for instance a viral infection, can trigger run-away intravascular thrombosis in patients with genetic mutations or neutralizing antibodies to thrombomodulin (298), ADAMTS13 (297, 302), or to complement inhibitors (293). Loss of sialic acid EC glycocalyx by pneumococcal derived neuraminidase can also trigger the hemolytic uremic syndrome in children (233), as can reduced VEGF signaling due to loss of function mutations in diacylglycerol kinase (291, 292). While albuminuria is common in patients with these disorders, end-organ damage due to microvascular thrombosis are clinically more significant.

### EC Glycocalyx Disruption in Critically ill Patients

Trauma, cardiovascular surgery, septic shock (303) and more recently in critical illness due to COVID-19 (7, 295), all are associated with generalized EC activation and EC glycocalyx disruption. While proteinuria is common in critically ill patients (6, 8), pulmonary and brain EC barrier disruption tend to have greater relevance for outcomes and therapy in these patients. Endothelial cell activation by inflammatory mediators, among them TNF-α and Il1-β, results in shedding of EC glycocalyx components exposing cell-surface adhesion molecules that enable the initial capture and rolling of leukocytes on the endothelium and integrin-dependent leukocyte transmigration (304). EC glycocalyx disruption also promotes platelet adhesion and reduces the anti-coagulant and fibrinolytic activity of the EC surface (305). Even so, a recent metanalysis concluded that while EC glycocalyx shedding is common in critically ill patients, it does not distinguish between various causes and is not consistently associated with “vascular leak” (116). Similarly, albuminuria in this setting is a non-specific marker of EC glycocalyx dysfunction.

## Summary and Future Considerations

The glomerular endothelium is a critically important component of the size- and charge-selective GCW barrier. Only a very small fraction of circulating albumin and other macromolecules can penetrate glomerular EC to reach the underlying GBM and sub-podocyte space. While glomerular EC fenestrae support filtration of massive volumes of water and small solutes, they are not permeable to larger plasma proteins due to a negatively charged, organized glycocalyx and ESL that covers the EC surface and fills the fenestrae. This pericellular environment not only serves as a physical barrier to macromolecules, it also controls the activity of many mediators, cytokines, growth factors, complement and coagulation cascades, and circulating cell and platelet repulsion/adhesion. Glycocalyx degradation in disorders that cause wide-spread EC dysfunction and/or activation, like the metabolic syndrome, diabetes, sepsis and other forms of systemic inflammation, result in glycocalyx degradation and proteinuria. More specific insults like VEGF pathway interruption and localized activation of complement and coagulation cascades can cause somewhat more restricted glomerular EC injury. Many components of the EC glycocalyx/ESL are known, but it is expected that there are unique aspects of its composition and organization in glomerular EC. To define these in health and disease represents a major, but important challenge for the future, given that most glycocalyx/ESL components are ubiquitous, and their function is not just defined by protein expression, but also by many position-specific polysaccharide modifications.

## Author Contributions

BB performed the literature review and wrote this manuscript. JN and BH have contributed fundamental research and insights to this topic and reviewed/critiqued the final manuscript. All authors contributed to the article and approved the submitted version.

## Funding

This work was funded by Principal Author's laboratory was from the Canadian Institutes of Health Research (##427186), the Natural Sciences and Engineering Research Council of Canada (#NSERC RGPIN-2016-05609), and the Heart and Stroke Foundation of Canada (#HSFC G-16-00013991).

## Conflict of Interest

The authors declare that the research was conducted in the absence of any commercial or financial relationships that could be construed as a potential conflict of interest.

## Publisher's Note

All claims expressed in this article are solely those of the authors and do not necessarily represent those of their affiliated organizations, or those of the publisher, the editors and the reviewers. Any product that may be evaluated in this article, or claim that may be made by its manufacturer, is not guaranteed or endorsed by the publisher.
