# Supplementary material for: The Glomerular Endothelium Restricts Albumin Filtration
Source: Front Med (Lausanne). 2021 Nov 29;8:766689. doi: 10.3389/fmed.2021.766689 (PMC8667033; doi:10.3389/fmed.2021.766689)
Supplement: Supplementary file 1 [file Table_1.DOCX]

**Table 1: Major Proteoglycans Produced by Endothelial Cells**

| **Protein Name** | **Gene Symbol** | **Core Protein MW** | **GAGs** | **Location** | **Function** |
| --- | --- | --- | --- | --- | --- |
| Syndecan-1 | SDC1 | 35 | HS & CS | Membrane-Spanning | All syndecans are expressed in Glomerular EC, As major components of the luminal glycocalyx, they contribute to the barrier function and signal EC remodeling in response to shear stress. Syndecan shedding reduces glyocalyx thickness and macromolecule barrier function. |
| Syndecan-2 | SDC2 | 22 | HS | Membrane-Spanning |  |
| Syndecan-3 | SDC3 | 46 | HS & CS | Membrane-Spanning |  |
| Syndecan-4 | SDC4 | 22 | HS | Membrane-Spanning |  |
|  |  |  |  |  |  |
| Glypican-1 | GPC 1 | 62 | HS | GPI-Anchored | The only glypican (of 6) expressed in EC,. Clusters in rafts. Mediates shear-stress  stimulated NO synthesis and is shed during inflammation. |
|  |  |  |  |  |  |
|  |  |  |  |  |  |
| Versican | VCAN | 373 | CS | Secreted, Hyalectin | Versican localizes to both, luminal and subendothelial glycocalyx. It specifically binds  Hyaluronan forming organized aggregates |
|  |  |  |  |  |  |
| Perlecan | HSPG2 | 468 | HS (In EC) | Secreted | Perlecan is a major component of ECM; It binds cell-surface integrins. May transmit  shear-stress signals at the luminal surface of EC |
|  |  |  |  |  |  |
| biglycan | BGN | 42 | CS/DS | Secreted, SLRPG | All small leucine-rich proteoglycans (SLRPG) are expressed by glomerular EC. They  localise to, and function in ECM organization. They also modulate growth factor activity. Lumican is part of the luminal glycocalyx. |
| decorin | DCN | 40 | CS/DS | Secreted, SLRPG |  |
| lumican | LUM | 39 | KS | Secreted, SLRPG |  |
|  |  |  |  |  |  |
| EC specific molecule-1  (Endocan) | ESM1 | 20 | CS | Secreted |  |
|  |  |  |  |  |  |
| Serglycin | SRGN | 20 | HS & CS | Intracellular | The only known intracellular granule-associated PG. |
| Betaglycan | TGFBR3 | 94 | HS & CS | Membrane-Spanning | Type III TGF-β receptor |
