# Supplementary material for: The Glomerular Endothelium Restricts Albumin Filtration
Source: Front Med (Lausanne). 2021 Nov 29;8:766689. doi: 10.3389/fmed.2021.766689 (PMC8667033; doi:10.3389/fmed.2021.766689)
Supplement: Supplementary file 2 [file Table_2.DOCX]

**Table 2: Major Sialoglycoproteins of the Endothelial Glycocalyx**

|  | **Protein Name** | **Gene Symbol** | **Core Protein MW** | **Glycosylation** | **Location** | **Function** |
| --- | --- | --- | --- | --- | --- | --- |
| **Sialomucins** | Podocalyxin | PODXL | 59 | 43 O-linked sialylated oligosaccharide  5 N-linked sialylated oligosaccharides | Membrane-Spanning | Restricted to the apical/luminal domain. Functions to repel other negatively charged surfaces/cells |
|  | Podocalyxin-like 2  (Endoglycan) | PODXL2 | 65 | 8 sialylated O-linked oligosaccharides | Membrane-Spanning | Restricted to the apical/luminal domain. Serves as a selectin |
|  |  |  |  | 2 N-linked oligosaccharides |  | ligand. Selectin binding requires oligosaccharide |
|  |  |  |  | 1 Chondroitin sulfate GAG |  | modification |
|  | Endomucin | EMCM | 27 | Highly O-glycosylated, sialic acid rich | Membrane-Spanning |  |
|  | CD34 | CD34 | 41 | 9 N-linked sialylated oligosaccharides | Membrane-Spanning | Marker of differentiated EC and hematopoietic and EC progenitors. Involved in homing of circulating prognenitor |
|  |  |  |  |  |  |  |
|  | Podoplanin | SDC4 | 17 |  | Membrane-Spanning | Restricted to lymphatic EC |
|  |  |  |  |  |  |  |
|  | Orosomucoid-1 | ORM1 | 24 | 5 N-linked sialylated oligosaccharides | Secreted, Circulating | Produced in the liver, orosomucoids are part of the ESL and enhance the EC barrier function. In homo sapiens ORM1,2 have been identified, mice also express protein from ORM3 |
|  | Orosomucoid-2 | ORM2 | 24 | 6 N-linked sialylated oligosaccharides | Secreted, Circulating |  |
|  | Orosomucoid-3 | ORM3 | 24 |  | Secreted, Circulating |  |
